# Development of a Connected Sensor System in Colorectal Surgery: User-Centered Design Case Study

**DOI:** 10.2196/31529

**Published:** 2022-07-08

**Authors:** Christel Schwartz-Lasfargues, Camille Roux-Gendron, Pim Edomskis, Isabelle Marque, Yves Bayon, Johan F Lange, Jean Luc Faucheron, Bertrand Trilling

**Affiliations:** 1 Public Health Department CHU Grenoble Alpes Grenoble France; 2 Inserm CIC 1406 Université Grenoble Alpes Grenoble France; 3 Floralis Grenoble Alps University Grenoble France; 4 Department of Surgery Erasmus Medical Centre Rotterdam Netherlands; 5 Medtronic – Sofradim Production Trevoux France; 6 Colorectal Surgery Unit Visceral Surgery and Acute Care Surgery Department CHU Grenoble Alpes Grenoble France; 7 TIMC Université Grenoble Alpes Centre National de Recherche Scientifique, Grenoble INP Grenoble France

**Keywords:** user-centered design, usability, formative evaluation, medical device, innovation, Internet of Things, IoT, colorectal surgery, colorectal anastomotic leakage, mobile phone

## Abstract

**Background:**

A successful innovative medical device is not only technically challenging to develop but must also be readily usable to be integrated into health care professionals’ daily practice. Through a user-centered design (UCD) approach, usability can be improved. However, this type of approach is not widely implemented from the early stages of medical device development.

**Objective:**

The case study presented here shows how UCD may be applied at the very early stage of the design of a disruptive medical device used in a complex hospital environment, while no functional device is available yet. The device under study is a connected sensor system to detect colorectal anastomotic leakage, the most detrimental complication following colorectal surgery, which has a high medical cost. We also aimed to provide usability guidelines for the initial design of other innovative medical devices.

**Methods:**

UCD was implemented by actively involving health care professionals and all the industrial partners of the project. The methodology was conducted in 2 European hospitals: Grenoble-Alpes University Hospital (France) and Erasmus Medical Center Rotterdam (the Netherlands). A total of 6 elective colorectal procedures and 5 ward shifts were observed. In total, 4 workshops were conducted with project partners and clinicians. A formative evaluation was performed based on 5 usability tests using nonfunctional prototype systems. The case study was completed within 12 months.

**Results:**

Functional specifications were defined for the various components of the medical device: device weight, size, design, device attachment, and display module. These specifications consider the future integration of the medical device into current clinical practice (for use in an operating room and patient follow-up inside the hospital) and interactions between surgeons, nurses, nurse assistants, and patients. By avoiding irrelevant technical development, this approach helps to promote cost-effective design.

**Conclusions:**

This paper presents the successful deployment over 12 months of a UCD methodology for the design of an innovative medical device during its early development phase. To help in reusing this methodology to design other innovative medical devices, we suggested best practices based on this case.

## Introduction

### Background

Developing a successful innovative medical device is a real challenge. In all, 3 dimensions must be considered: technical aspects, regulatory framework, and usability of the device. To be adopted, the medical device must be usable, useful, satisfying, and safe for future users. Furthermore, medical devices should not only address unmet clinical needs but also provide demonstrable benefits for patients. The industrial development must also be cost-effective, to avoid jeopardizing the commercial launch and economic viability of the device.

The technical dimension of development is essential to obtain a functional device. The initial stages naturally often focus on and devote efforts to these aspects.

The regulatory dimension is complex owing to the multiple specificities of each project. Full compliance with the European regulations relating to medical devices (European Union Medical Device Regulation 2017/745) is required for the device to enter the market. In the early phases of development, ergonomic features are often understudied. These features are essential to ensure the safety of the medical device for all users. Compliance with the European regulations is presumed when the standard *Application of usability engineering to medical devices* (International Electrotechnical Commission [IEC] 62366-1:2015) is followed.

A user-centered design (UCD) approach aims to place future users at the center of the design process. Benefits of this approach are described in Textbox 1. Co-design is managed by combining technical and user expertise, including cycles with a requirement analysis phase; a design, test, and realization phase; and an implementation and monitoring phase [1]. The entire cycle or specific phases can be repeated until the objectives are achieved to the satisfaction of all the stakeholders.

**Benefits of a user-centered design approach.**

- Adoption—when all potential users are involved from the early design phases, clinical adoption of the device is generally much high [2].
- Better adaptation of the device to future users’ needs [3-5].
- Better engagement between users, designers, and other stakeholders [6].
- Better communication regarding design [6].
- Heightened dynamics—“The iterative nature of user-centered design means that assumptions are continually challenged and revised throughout the development process. This means the perspectives of team members evolved throughout the project as more information was uncovered and incorporated” [7].
- Economic gain—lack of use analysis is a cause of budget underestimation for information technology and health projects [8,9]. “An investment in usability testing can benefit manufacturers in myriad ways, including optimising development schedules, increasing sales, simplifying training and product support, and reducing legal exposure” [10].

This paper presents a case study illustrating the integration of the usability dimension from the early design phase in the development of an innovative medical device, with the aim of reducing costs and avoiding slowing down of the development phase between concept and proof of concept.

### Study Context

This case study is related to the design of a device to monitor postsurgical complications following colorectal surgery.

Abdominal surgery is continuously improving owing to the development of new surgical procedures, particularly, minimally invasive techniques such as laparoscopy and robot-assisted surgery. In addition, patient care has evolved with fast-track management [11,12]. Despite these significant advances, complications continue to occur. The most detrimental postsurgical complication following colorectal surgery is colorectal anastomotic leakage (CAL). CAL occurs frequently and can be serious, with incidence varying between 3% and 19% [13-17] and mortality rates between 2% and 18% [14,16,18,19]. Moreover, CAL remains challenging to detect at an early stage. On average, CAL is detected 7 to 9 days after surgery [20,21], when the patient may be recovering at home. A total of 18% of cases of CAL are diagnosed after the patient has been discharged from the hospital [22]. This situation can be problematic, as delay of 2.5 days in diagnosis has been linked to increase in mortality from 24% to 39% [23]. Consequently, early detection of CAL is key to improving postoperative outcome for patients.

One of the main challenges is to identify biomarkers that predict CAL. An innovative strategy involves detecting biomarkers in wound exudates collected from an intra-abdominal drain. The identification of the most relevant, indirect, and predictive markers of infection has been previously studied by the present authors and collaborators [24,25]. These studies involved 540 patients who were treated for colorectal resection and underwent colorectal surgery between 2007 and 2018. On the basis of the good specificity and selectivity of the combination of pH and lactate, both biomarkers have been selected as the most promising candidates [26,27] because changes in the levels of these biomarkers in real time could help to correctly monitor the onset of CAL and modify therapeutic strategies. To meet the challenge of monitoring these biomarkers, a breakthrough innovation was considered: a smart sensor system connected to the drain, deployed and activated at the end of the surgery. This system would provide early alerts to adapt patient care immediately upon the detection of biomarkers of concern.

### Presentation of the Connected Sensor System

**Figure 1:**
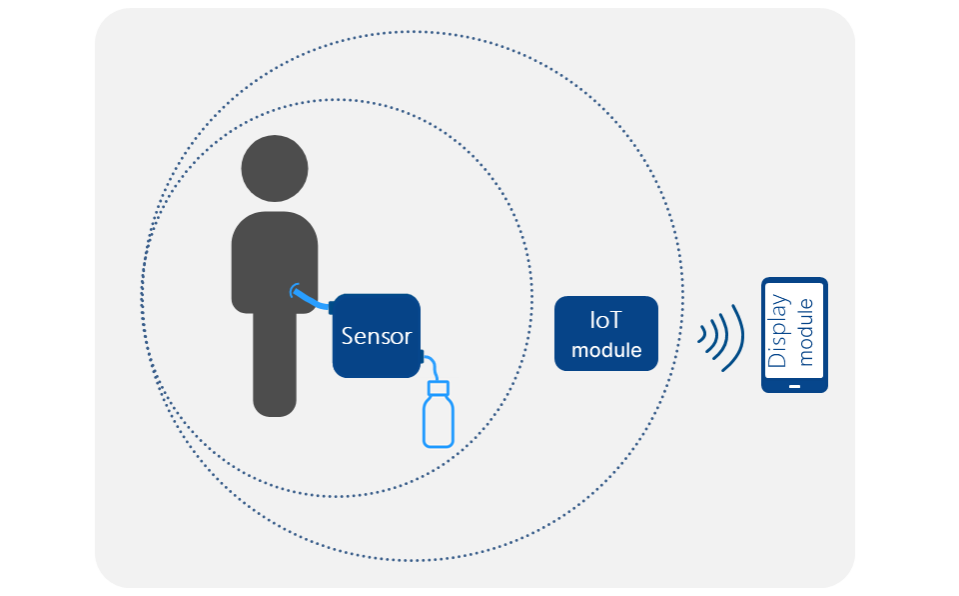
Overview of the Exucheck solution system. IoT: Internet of Things.

The Exucheck project was designed to develop a system that will provide early alerts for postoperative infections, for example, anastomotic leakage following colorectal surgery or infected hematomas. The system is based on a sensor device that analyzes wound exudate following abdominal surgery. Exudate or peritoneal fluid is routinely collected using a drain placed during surgery for rectal cancer. The obtained exudate can be analyzed by the sensor system in real time. The additional data generated can alert nurses and physicians to infection, even before clinical signs become observable. The medical device is composed of 3 components (Textbox 2; Figure 1).

**Components of the medical device.**

- A sensor module is connected to the patient’s drain. The exudate passes through the measuring chamber in the sensor module. It is set up by health care professionals.
- A reusable Internet of Things module or communication board is clipped to the sensor module, converting it into a wireless communicative medical device. It is set up by health care professionals.
- A display module shows all the information transmitted and computed from the data generated by the sensor and Internet of Things modules. It is used by health care professionals.

Once the sensor and Internet of Things (IoT) module is connected to the patient’s drain by the health care professionals in the operating room (OR), this unit remains attached to the patient during their hospital stay (approximately 3-5 days until discharge).

In the OR, after surgery, the surgeon will connect the sensor module to the drain. Then, the IoT module is connected to the sensor module. The setup is done using the display module. Regularly (configurable timing), the IoT module retrieves the information of pH and lactate values measured by the sensor module. The staff uses a dedicated smartphone app to assess the sensor values on the display module. One display module can follow several patients and can be shared among health care professionals. The patient is not expected to interact with either the device or the display module.

### Project Partners

All the partners were involved in the device design (Table 1); they incorporated their own requirements (electronic, electrochemical, and industrialization) and the clinical and patient needs into the design process.

**Table 1:** Project partners and their roles.

Partner	Type	Role and expertise
Medtronic	Industrial	Project leader and MedTech expertise (industrialization process and business)
Maatel	Industrial	Electronic and IoT^a^ expertise and design and develop the IoT module
CEA^b^	Academic	Electrochemical sensor expertise and design and develop the lactate sensor
Grenoble-Alpes University Hospital	Academic	Clinician expertise and formative evaluation
Erasmus Medical Center	Academic	Clinician expertise and formative evaluation
Grenoble-Alpes University	Academic	UCD^c^ expertise

^a^IoT: Internet of Things.

^b^CEA: Commissariat à l’énergie atomique et aux énergies alternatives.

^c^UCD: user-centered design.

## Methods

### Ethics Approval

All participants in Rotterdam provided oral consent and participated voluntarily without a dependency situation. Ethical approval was not sought in the Netherlands for this study, as it is not subject to the Medical Research Involving Human Subjects Act (Wet Medisch-Wetenschappelijk Onderzoek met Mensen [28]), according to the guidelines of the Erasmus Medical Center’s ethical committee (Medisch Ethische Toetsings Commissie [29]) and the Netherlands’ national legislation [30].

All participants in Grenoble provided oral consent. Ethical approval was not sought in France. As a noninterventional human factors study, which posed minimal risk to the participants, this study was deemed to fall outside the scope of the Jardé Law [31].

### A Cooperative Design Methodology

To develop this system, a UCD approach was implemented as described in standard International Standards Organization 9241-210 and standard IEC 62366-1:2015. The process required all design choices to be systematically assessed by the users.

For this case study, a *cooperative design* methodology was chosen to benefit from the active participation of future users alongside other stakeholders. This methodology allowed future users to actively and creatively participate in device design [32].

**Figure 2:**
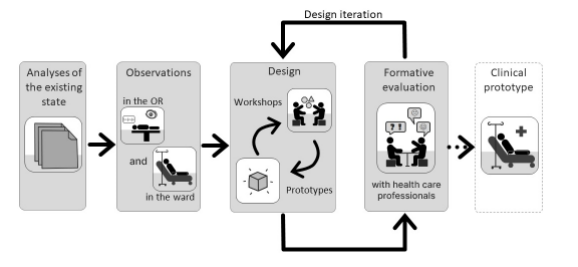
Outline of the methodology used—designed by the authors. OR: operating room.

In the cognitive ergonomics literature, many possible methods have been proposed to integrate users and designers in the dynamic process of system development [32]. The following data collection techniques were implemented here (as described in Figure 2): observations, participatory design through workshops (with Medtronic, Maatel,

Commissariat à l’énergie atomique et aux énergies alternatives, and Grenoble-Alpes University), and formative evaluation through user tests (with Grenoble-Alpes University Hospital Erasmus and Grenoble-Alpes University).

This methodology was implemented from the beginning of the project, in parallel with the technical development of the device. The specificity of our intervention was that a first iterative loop (observation, design, and formative evaluation) was completed before producing a functional prototype system.

This methodology aimed to address 2 important challenges at this stage of the project.

As the users of this device are potentially numerous (surgeons, OR nurses, and department nurses), the first challenge was to define how and when the different stakeholders could interact with the system, from the OR till the patient’s discharge. The system should be smoothly integrated into the daily clinical practice.

The second challenge was to propose technical characteristics for the development of the system, such as the size, weight, and attachment system on the patient.

### Analyses of the Existing State (January 2019 to February 2019)

Analyzing the overall background of the industrial partner’s project shed light on the key points to be addressed:

1. Commercial: market and competitive landscape study
2. Technical: work on biomarkers [24-27] and development of connected pH and lactic acid sensors
3. Use of the device: initial feedback from expert health professionals (voice of the customer) on the clinical needs and means to address them and storyboard presenting how the device will be used by experts
4. Literature review

From the first clinical needs identified, these documents helped to define a strategy for the technical deployment and wide adoption of the device, considering the future users and how the new device can be integrated into their practice. The knowledge at the beginning of the project was mainly provided by experts, without fully integrating the realities in the hospital setting. The initial focus of this case study was interest in the system, rather than its real future use and the corresponding challenges.

Thus, close observation of the actual work of future users should provide valuable knowledge, serving as a basis for the whole UCD.

### Observations (March 2019 to May 2019)

In all, 2 European hospitals were recruited as partners in the project, the Erasmus Medical Center in Rotterdam (the Netherlands) and Grenoble-Alpes University Hospital (France), to include complementary dimensions in the project (different countries and organizations; involvement in similar projects). Moreover, Erasmus Medical Center was involved in 2 clinical studies with Medtronic to determine the interest in biomarker use [24,32], and Grenoble-Alpes University Hospital is intensively committed to evaluate innovative medical devices, with a group entirely comprising experts in UCD approaches. As cultural and organizational differences were expected, observations were conducted at both sites. In Grenoble, observations were conducted by 2 usability engineers. In Rotterdam, they were performed by a usability engineer, a French surgeon, and a Dutch physician.

Several visits were made to observe routine practices and analyze the work of the various participants (surgeon, nurse manager, nurse, and nurse assistant in the operating theater and hospital ward). A total of 5 elective procedures (laparoscopy and laparotomy) were observed, 1 at Erasmus Medical Center and 4 at the Grenoble-Alpes University Hospital. Totally, 5 shifts (morning, afternoon, and night shifts) were observed in the digestive ward of the Grenoble-Alpes University Hospital. In addition to the data gathered during the course of these observations, interviews were conducted with several selected users.

The objective of these observations was to understand the use context, tasks to be completed, tools currently used, and how conditions vary from day to day. These observation times highlighted the needs and expectations of the target users and the potential difficulties and constraints they may face. This information was used to design a tool that would be easy to integrate into the health care professionals’ daily practice.

### Workshops (June 2019 to October 2019 and February 2020)

Participative workshops with members of the development team were conducted to promote an efficient design of the medical device. They were facilitated by the presence of 2 usability engineers involved in the project. Industrial partners provided complementary expertise with new and integrative ideas on the development of the system [33]: Maatel for the development of the IoT module, Commissariat à l’énergie atomique et aux énergies alternatives for the development of the sensor module, Medtronic for the global vision and commercial strategy of the project, and usability engineers for the integration of the users’ needs.

A total of 4 themed workshops were set up in the Grenoble area. The sessions were centered around the manipulation and handling of the device in simulated condition. Thus, a life-size mannequin’s bust and several drains were available in each session.

The size and weight of the device were the subject of the first workshop. To ensure the efficacy of the session, a mock-up of the future device, electronic components similar to those that will be used in the future device, a balance, and a ruler were available. The manipulation of the objects provided support to overcome the unavoidable technical constraints regarding the needs of the users. Through exchanges and proposals, the technically viable size and weight were determined.

The first version of the device was 3D printed with the previously defined size and weight.

The second workshop was centered on connectivity between the sensor and the IoT modules. Each member manipulated different connection systems previously identified by the usability engineers. The session ended with the selection of a connection system that was both easy to handle, sufficiently safe to avoid involuntary disconnection of the 2 modules, and technically feasible.

The wireframe smartphone interface was the theme of the third workshop. The first version was created by the usability engineers using Adobe Experience Design (Adobe). The aim was to test the interface through different scenarios to readjust the wireframe in preparation for user tests.

The final workshop was conducted after the usability tests (refer to the *Formative Evaluation Through Usability Tests* section) and focused on the device’s attachment system to the patient.

To affix the system, a technical constraint was the length of the drain. The device should be placed at 10 cm from the abdomen (to avoid degradation of exudates along the drain), which limits the options for positioning the sensor module. As the system has significant weight, the risk of it pulling the sutures holding the drain in place as it exits the skin (major source of pain and infection) must be considered. Therefore, it is necessary to be able to attach it to the patient. During this workshop, attachment systems used on stoma and urinary pouch, camera pouch, and belt pouch were considered and manipulated to converge on an ideal solution.

Working with low-level equipment has the advantage of allowing the user to visualize how the device may be integrated into their daily practice without hindering imagination. Using systems that seem very mature or products that require high developmental cost often hinders creativity. These workshops promoted the rapid development of a prototype system, which was tested with the expected users to obtain data from the clinical field.

### Formative Evaluation Through Usability Tests (November 2019 to December 2019)

A total of 5 usability tests were organized with 13 participants: 3 groups with nurses and nurse assistants (n=2, 67% in Grenoble and n=1, 33% in Rotterdam) and 2 groups with surgeons and surgical residents (n=1, 50% in Grenoble and n=1, 50% in Rotterdam), to compare views and analyze feedbacks. These tests were conducted by 2 usability engineers.

Tests were performed using low-level mock-ups (3D-printed model). They were sufficient at this stage of the study and perfectly allowed users to project themselves to future use of the tool. Thus, simple technical designs were developed, not going as far as anything sufficiently tangible to be presented as functional to future users.

**Figure 3:**
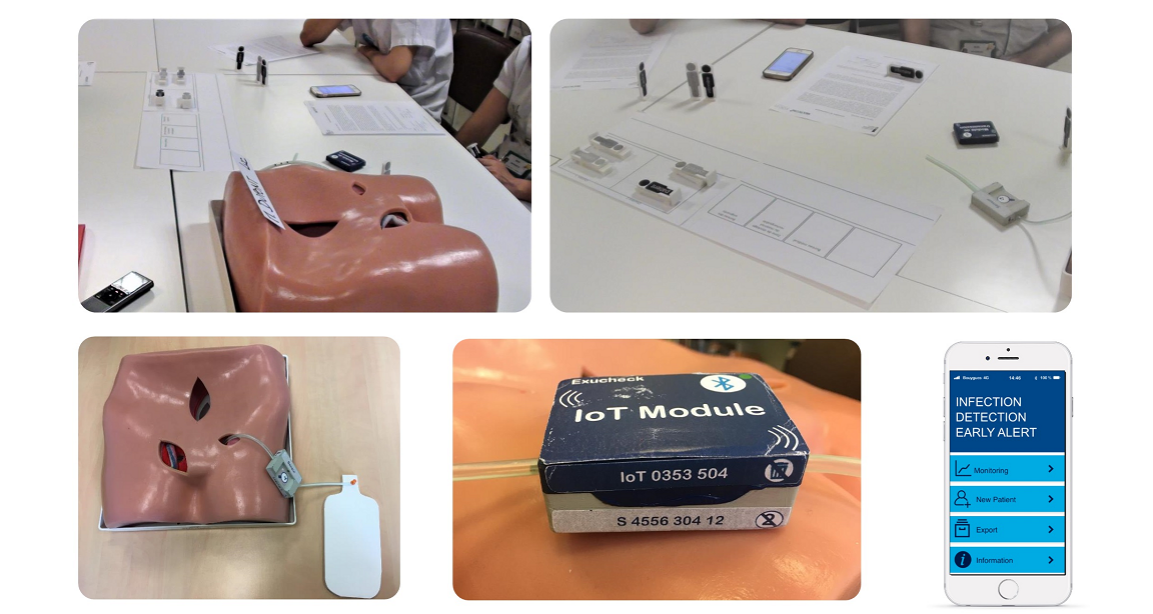
Usability test setup.

To conduct the tests as efficiently as possible, a transportable *test kit* (Figure 3) was developed in addition to the mock-ups and wireframe smartphone interface. This kit included the test protocols, a camera, and a mannequin bust.

The aim of the Exucheck project was explained to all the participants before the start of the usability tests. Participants were free to ask questions, and all questions were answered. During the test, participants were asked to perform a set of tasks that they may be asked to perform in the future, using the whole prototype system (sensor, IoT modules, and wireframe smartphone interface). For example, they were instructed as follows:

The patient (Mrs. de Groot Ann) is coming out of surgery. A drain was placed after the surgery. To analyse the exudates, the surgeon placed an “Exucheck” sensor on the drain. You must now plug the IoT module into the sensor in order to get the exudate analysis. You have a Smartphone that will guide you through the different steps to install the IoT module. It's your turn now!

Participants could stop any suggested task during the usability test. It was emphasized that it was the Exucheck system that was tested, not the participants. All participants provided their consent to participate in the usability tests and have the session recorded by video and audio for subsequent analysis.

The participants were able to comment on the size, shape, and weight of the mock-ups and the ease of navigation on the wireframe smartphone app.

User tests made it possible to do the following:

1. Present the concept to future users.
2. Gather early feedback to identify points that are not yet addressed.
3. Confirm or better understand clinical practice regarding the introduction of the system.
4. Identify cultural differences between Dutch and French practices.
5. Propose improvements to the system.

## Results

### Context in Which the Device Is Used

Field observations allow better understanding of the tasks, interactions, and constraints associated with the context in which the new system will be integrated. The aim of this case study was to propose transparent and seamless integration of the system into daily practice.

We initially assumed that the device would be fully deployed at the end of colorectal surgery. However, the end of a surgery corresponds to a phase in which the OR nurses are particularly busy. Each additional task linked to the introduction of this system can delay the operating schedule, which is undesirable for the just-in-time organization of the operating theater. Thus, another setup was proposed: only the sensor module must be calibrated in the OR. When the patient returns to the ward after surgery, a nurse and nurse assistant round occurs. Then, a few additional minutes can be taken to attach and activate the IoT module.

During hospitalization, and consistent with real hospital organization, the best time to monitor the sensor data is as part of standard nurse and nurse assistant rounds, when other biological parameters are recorded (temperature, blood pressure, etc). Every minute is accounted for in a hospital environment. Over the course of observations and interviews, the fear of adding to the workload was mentioned several times. Thus, it is essential that the tool can be configured as quickly and automatically as possible. Owing to the many daily constraints faced by hospital staff, this device should not be seen as an additional hurdle.

Daily practices were not significantly different in terms of patient trajectory of care between the Grenoble-Alpes University Hospital and Erasmus Medical Center. However, 2 main distinctions were observed: lower frequency of nurse rounds in Rotterdam than in Grenoble and the role of a physician assistant to whom the nurses refer in Rotterdam. This role does not exist in France. However, at this stage of the project, these differences do not affect the integration of the medical device into daily clinical practice.

The results of this observation phase are valuable as they represent a rich source of information to refer to whenever a change is made to the product over the course of its design.

To conclude, the workflow was enriched and validated through the usability tests. The medical device was considered compatible with the normal daily practice and workload of health care professionals.

### Features of the Exucheck System

The overall feedback on the features of the system was good from all participants; they expressed interest in the concept.

Most future users were willing to test it (“When do you think it will be available for clinical tests?” [surgeon]) and found the concept easy to use (“Good. It is intuitive. It is not complicated”).

Now, we present the features of each element of the system.

### The Sensor and IoT Modules

**Figure 4:**
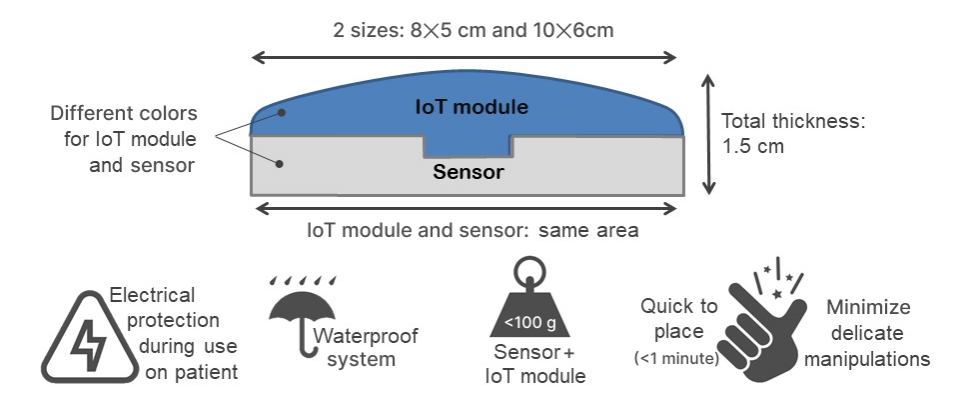
Main specifications of the sensor and Internet of Things (IoT) modules system.

The following recommendations emerged from dedicated workshops and usability tests (Figure 4).

As the weight (maximum 100 g) of the device is a technical constraint (sum of the weight of the electronic board and estimated weight of the sensor module), the perception of load was studied. This perception depends on the density of the device. Thus, the density can be modified to evenly distribute the weight, to reduce the impression of carrying a load.

**Figure 5:**
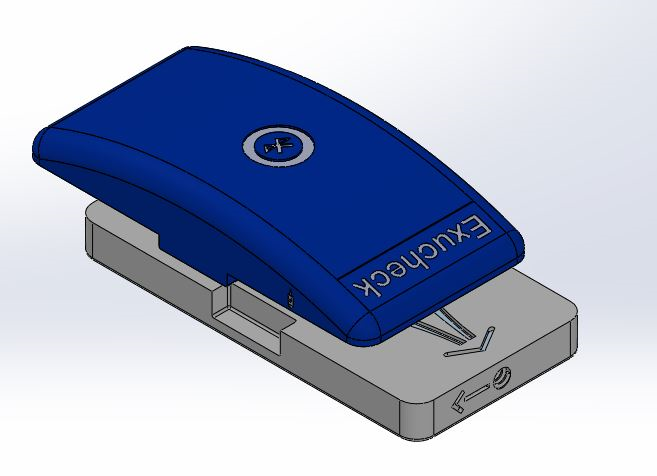
Illustration showing the curved design.

The final design that was selected was curved (Figure 5) to reduce the impression of bulkiness that users mentioned about the previous 3D model (a parallelepiped).

Regarding the connection between the two modules, the participatory design led to the following:

1. Mechanical connection was made through the lateral buttons. Magnet will be used. Push buttons were recommended for the final design.
2. Electrical connection was made through a pin in the IoT module that activates the sensor module when the IoT module is plugged onto the sensor module.

### Device Attachment

The main technical constraint for attaching the system was the length of the drain placed at 10 cm from the abdomen. Usability tests revealed the criticality of this issue, with differing views among the nurses, nurses’ assistants, and physicians on *where and how* to affix the device. The workshop dedicated to this aspect also did not result in consensus on a potential placement and fixation of the system. It was concluded that more technical tests were required to remove certain constraints and ensure that the device met the practitioners’ requirements.

**Figure 6:**
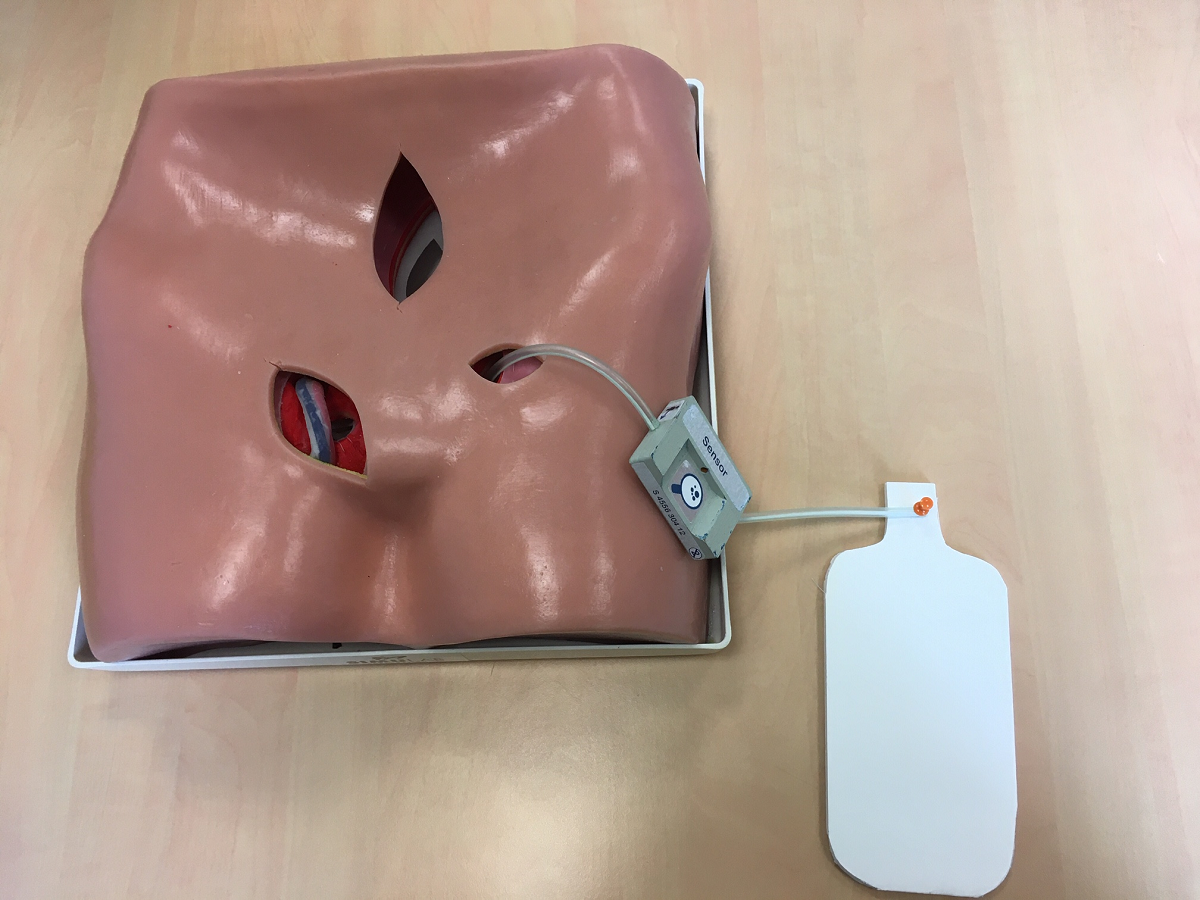
Illustration showing the issue regarding placement of the device on the drain attached to the patient’s abdomen.

Thus, how the system should be placed on the patient remains to be resolved. The following key parameters were identified: diameter and length (10 cm) of the drain between the sensor module and where the drain exits the skin (Figure 6). To progress on resolving this question, a functional prototype system is required. Currently, two strategies can be envisaged:

1. Seeking solutions and continuing the design iteration cycles. This strategy will allow clinical studies to begin with a more complete device that is close to the commercial device. The clinical study can be used as a support for the summative evaluation to complete the usability file for European Conformity marking [34].
2. Pursuing the proof of concept with a clinical study, despite this issue. This strategy will allow verification of the technical efficacy of the system. Furthermore, it can be used to identify key points to choose the most suitable solution. For example, if the measurements at 10 cm and 50 cm from the exit through the skin are similarly reliable, the sensor and IoT modules can be placed further from the patient’s abdomen. This possibility will allow great freedom on where to place the system.

### The Display Module

**Figure 7:**
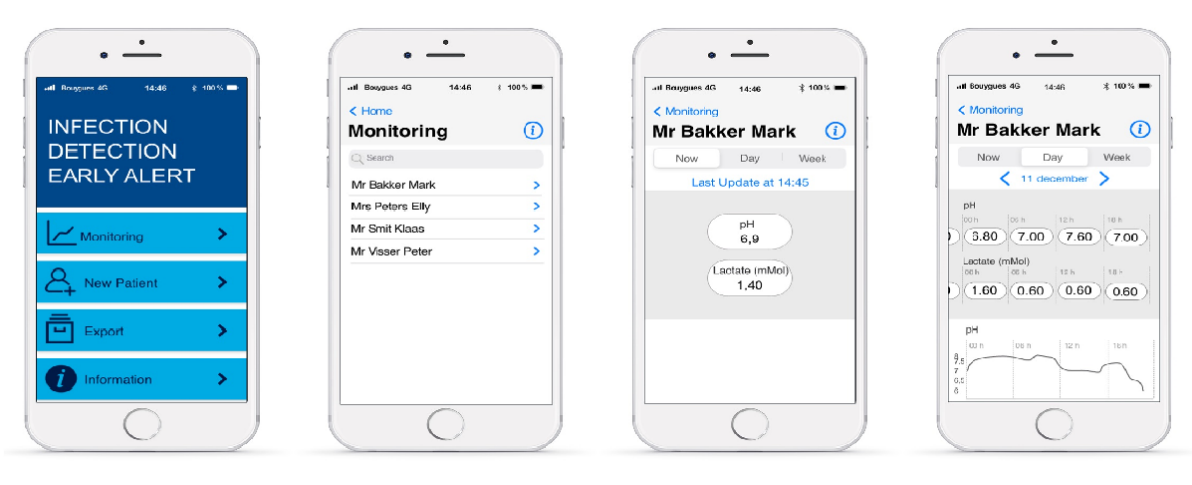
Wireframe smartphone interface for the display module.

A wireframe interface of the mobile app was created using Adobe Experience Design (Figure 7).

Over the course of discussions with nurses, issues with smartphone type, screen size, security, robustness, and risk of theft were discussed.

Regarding the mobile app, the feedback was positive, indicating that the handling was intuitive, even for professionals who are not particularly technically aware. However, it was recommended that the font size be increased and access to the results be simplified by minimizing the number of clicks or even eliminating them. For example, the staff suggested that simply bringing the phone close to the sensor and IoT modules should trigger the display of results.

Regarding the use of a medical device based on a smartphone, the health care professionals are open to the idea of having a small touch-sensitive device. The main fear was theft or damage of the device. Therefore, it is important that it is robust and not very attractive to avoid being stolen.

## Discussion

### Strengths of the Study

The implemented methodology, through observations, workshops, and usability tests, contributed to resolve the 2 challenges (integration into the daily practice and technical features) raised at the beginning of the study. The UCD enabled us to converge toward a common vision of a solution that best meets the requirements of the field and the technical constraints. At the end of this first stage of the project, we obtained a set of recommendations. This medical device will be developed according to feedback from technical tests and the next feedback from users.

The importance of implementing a UCD approach as early as possible in an innovative project was largely confirmed by the results obtained from this case study. Key points to be considered when working on a medical device are discussed below.

First, a UCD implemented at the earliest stage in the development of a medical device makes it possible to guide the design and plan the new technology such that it is consistent with the various users’ (physician, surgeon, nurse, etc) daily practices, almost from the beginning. This type of approach guides toward a solution that will be easy to integrate into current care practices in an abdominal surgery department, while remaining technically feasible. Meetings and discussions with future users as early as possible ensure that strategic decisions are made appropriately, such as relating to the shape of the system and its operating mode. Beginning with technical developments can be counterproductive (loss of time and money) if they are not adapted to the reality in the field. By considering the end users’ feedback from the earliest stages of the design process, it becomes possible to develop robust specifications for the technical partners within a limited time frame. The cycle of observations, workshops, tests, and new versions of the prototype systems was completed within 12 months, before the culmination of technical development.

Second, the techniques and tools selected were relevant to the context of this medical device and the time frame of the project.

In particular, when designing a medical device for use in a hospital environment, it is important to consider the entire life cycle of the system. In our case, observations began when the device was implemented during abdominal surgery (ie, preparation of the OR) and were continued till the patient was discharged from the hospital. Observation of all the steps allowed a systemic view of the patient’s trajectory and helped to understand how the system can be integrated into the care trajectory. Multiple interactions around the patient were noted, which allowed us to envisage how the system will be managed by the distinct groups of health care professionals.

Workshops organized with industrial project partners created a real, dynamic environment around product design. For the case presented here, each workshop was dedicated to a specific developmental aspect: shape of the system, attachment system, and so on, making them attractive, concise, and effective. Each member of the project brought their own expertise based on their field experience and shed light on the expectations of future users based on the data collected during the observation phase. The workshop format was found to be particularly efficient. A wealth of results was obtained over a short period with minimal financial investment. Moreover, by remaining in touch with the needs of end users—medical staff and patients—the team maintained a realistic approach to the system’s design, which gave great meaning to the project. Thus, this approach quickly reinforced synergies between partners by decompartmentalizing expertise.

From the beginning of the project, using low-level material made it easy to illustrate what was being said and develop the concept. In our project, a simple 3D print, a drain, and a mannequin bust allowed good projection, for both the future users and the project team. Touching and manipulating elements makes the experience more tangible and generates very rich feedback. In the early stages of a project, functional prototype systems are not necessary or even recommended. Proposing *handmade* prototype systems encourages users to modify them and propose improvements.

Finally, user involvement is central to a UCD approach and requires appropriate strategies for each situation and project, particularly, in a hospital environment. Significant responsibility is involved, as patients’ health depends on the work of the health care staff. Moreover, the health care team’s time is precious, and their availability depends on their working hours, which are often staggered. To adapt to these constraints, it is necessary to do the following:

1. Anticipate the time and financial resources needed to facilitate the participation of nurses and physicians [10]. As this case study was conducted as part of the Exucheck project, associating the health care professionals as partners in the definition of the project was key to their involvement.
2. Be available. This made it possible to conduct observations throughout the nurses’ and physicians’ work, regardless of their working hours. When integrating a new medical device into a department, the atmosphere and tasks differ over the 24-hour period; this can have an impact on how a device is used and on nurses’ and physicians’ interactions with the system. By appointing 2 usability engineers to work together on the project, a large number of situations could be observed and the results could be compared.
3. Be reactive. An evaluation kit that was always ready and transportable was an asset when conducting user tests. This kit allowed rapid access to the field to exchange views with the team on a last-minute slot and bring the device evaluation tools as close as possible to the nurses’ and physicians’ workplace.
4. Adapt the organization of user tests. Nurse–nurse assistant pairs were involved, which helped their projection into conceptual innovations. As a result of
  shared awareness
  [35] between users, new ideas were more forthcoming and converged more rapidly toward a solution through a limited number of tests. A clinical team should share their practices to help in designing technologies for the team. However, bias can occur as a result of hierarchical relationships within the team conducting tests. The usability engineers must remain vigilant during tests to ensure that they are gathering all points of view.

### Limitations

One of the recognized limitations of UCD is that it considers only end user feedback for design choices and addresses technical constraints in parallel. The co-design approach that we used enabled us to remain as vigilant as possible on this point and to continually confront the needs and expectations of users regarding the technical constraints. However, we note that on certain aspects, notably the attachment of the system to the patient, our methodology has not yet succeeded in proposing a convincing solution. This shows that the user does not always have answers to the problems and that the proposed solutions are sometimes technically unfeasible. Therefore, it is important to set up new design loops involving the end user and the technical team in the design choices. This is especially true as users can evolve, change their minds, gain expertise, and transform their practice. Therefore, our role is to keep a critical eye on their feedback and be open to any request for change. It is also important to bear in mind that every technological advance can call an initial user need into question and vice versa.

Although patients are end users of the medical device, they were not consulted for this project. This was a conscious decision related to the level of progress of the development. In this first phase, the daily life of the patients was observed and analyzed in concertation with nurses and physicians. When a functional system is developed, feedback from patients will be essential. At this stage, there were several questions about the system and how it works to take full advantage of patient feedback.

Another limitation was that observations at both sites occurred only during elective surgical interventions. Thus, the results and recommendations do not consider the specificities of emergency surgery.

The study did not mention criteria such as safety (for the patient and health professional), sterilization or disinfection requirements, biocompatibility materials, antireflux system, and so on. By mutual agreement between the partners, these points have not been considered as priority in this early phase of the project and will be analyzed at a later stage in the mandatory process of risk analysis linked to usability engineering, when the first prototype is available.

### Conclusions

This paper presents the deployment over 12 months of a UCD methodology for the design of an innovative medical device during its early development phase.

The approach was implemented at the beginning of the project, from the concept of the medical device, and in parallel to overcoming technical barriers. The advantages of integrating the usability of the device during this step and in close collaboration with the technical teams, notably through workshops, were the following:

1. Identify valuable technical features (eg, shape, size, and weight) for hardware integration of the sensor module, IoT module, and software interface and avoid unnecessary generation of functional hardware that fails to meet needs. This approach promotes cost-effective development with minimized iterations.
2. Rapidly counterbalance user constraints with technical solutions.
3. Gather initial positive indications of future adoption of the system. This encourages continued technical development and financial investment.

The key to the success of our case study was the techniques implemented (observations, workshops, and usability tests); our adaptation to collecting user feedback in a hospital environment with constraints, particularly in terms of the availability of nursing staff; and finally, the active and enthusiastic involvement of the project team. The interest of the whole team in the results of the usability study and in the technical advances also facilitated their appropriation and integration over the course of the project.

The results show that involving various stakeholders around the notion of usability from the beginning of a project is possible through the implementation of immersive and collaborative techniques and through the very early manipulation of nonfunctional prototype systems.

The targeted objective was achieved. Technical and functional specifications were obtained within a few months. The UCD approach did not hamper technical development and, in contrast, optimized and enriched the development directions. The specifications consider future uses, needs of professionals, and technical constraints, while also facilitating future device integration within the hospital and as part of the patients’ trajectory.

This study presents the first iterative loop within the framework of a UCD approach. This starts the usability engineering file according to standard IEC 62366-1:2015 to ensure compliance with European Regulation 2017/45. The approach will support the continuation of the project through other iterations until the final version of the system is produced.

## Abbreviations

CAL: colorectal anastomotic leakage
IEC: International Electrotechnical Commission
IoT: Internet of Things
OR: operating room
UCD: user-centered design
